# Psychological characteristics and coping strategies of affected others of patients with gambling disorder

**DOI:** 10.3389/fpsyg.2026.1837244

**Published:** 2026-06-19

**Authors:** Elias Guillén-Guzmán, Amparo Del Pino-Gutierrez, Roser Granero, Tomás Santalucia, Ester Codina, Fernando Fernandez-Aranda, Eduardo Valenciano-Mendoza, Bernat Mora Maltas, Joel Tremblay, Aida Romero, Imma Borjabad-Fraga, Lia-Tamar Sánchez-Salido, Lorena De Blas-Soto, Susana Jiménez-Murcia

**Affiliations:** 1 Doctoral Programme in Medicine and Translational Research Research Line: 101275 – Clinical and Experimental Neurosciences, University of Barcelona, Barcelona, Spain; 2 Department of Child and Adolescent Psychiatry and Psychology, Institute of Neurosciences, Hospital Clinic Barcelona, Barcelona, Spain; 3 Department of Public Health, Mental Health and Maternal and Child Health Nursing, School of Nursing, University of Barcelona, Barcelona, Spain; 4 Psychoneurobiology of Eating and Addictive Behaviors Group, Bellvitge Biomedical Research Institute (IDIBELL), Barcelona, Spain; 5 CIBER Physiopathology of Obesity and Nutrition (CIBERobn), Health Institute Carlos III, Barcelona, Spain; 6 Department of Psychobiology and Methodology, Autonomous University of Barcelona, Barcelona, Spain; 7 Department of Fundamental and Clinical Nursing, School of Nursing, University of Barcelona, Barcelona, Spain; 8 Adult Mental Health Centre Guinardó, Horta-Guinardó Mental Health Association, Barcelona, Spain; 9 Clinical Psychology Department, Bellvitge University Hospital, Barcelona, Spain; 10 Department of Clinical Sciences, School of Medicine and Health Sciences, University of Barcelona, Barcelona, Spain; 11 Care and Research in Socio-Addictions (AIS), Mental Health and Addictions Network, Government of Catalonia, Comprehensive Public Health System Use Network of Catalonia (SISCAT), Barcelona, Spain; 12 Departament of Mental Health and Addictions, Consorci Sanitari del Maresme, Mataró, Spain; 13 Department of Psychoeducation, Université du Québec à Trois-Rivières, Trois-Rivières, QC, Canada; 14 Department of Psychiatry, Hospital de Sagunto, Sagunto, Spain

**Keywords:** affected others, coping strategies, gambling disorder, gambling-related harm, psychological distress

## Abstract

**Introduction:**

Gambling disorder generates substantial psychological, social, and economic harm not only for affected individuals but also for the relatives who support them. However, evidence regarding the psychological burden and coping strategies of these Affected Others (AOs) remains limited. This study aimed to examine the sociodemographic and clinical characteristics of patients receiving treatment for gambling disorder and their AOs, compare differences according to family relationship type and gender, analyze associations between coping strategies and psychological distress among AOs, and explore predictors of global psychological distress in this population.

**Methods:**

A total of 184 patients diagnosed with gambling disorder according to DSM-5 criteria and their respective AOs participated in the study. Participants completed structured interviews and validated psychological assessments evaluating sociodemographic characteristics, coping strategies, impulsivity, and psychological distress.

**Results:**

AOs were predominantly women in partner or parental roles and reported high levels of psychological distress. Partners showed poorer mental health profiles, whereas fathers were more frequently older and unemployed. Across the sample, maladaptive coping strategies, particularly self-criticism and social withdrawal, were strongly associated with higher psychological distress, while problem-solving strategies were related to better emotional well-being. Regression analyses identified coping strategies, patient impulsivity, and patient self-related variables as significant predictors of AO psychological distress, explaining approximately 30% of its variance.

**Discussion:**

These findings highlight the substantial emotional impact experienced by AOs of individuals with gambling disorder and emphasize the protective role of adaptive coping strategies in their mental health. Interventions aimed at strengthening problem-solving abilities, promoting healthier emotional expression, and reducing maladaptive coping responses such as self-criticism and isolation may improve relatives’ well-being and contribute to more comprehensive and effective treatment approaches for gambling disorder.

## 1 Introduction

Gambling Disorder (GD) is a behavioral addiction associated with substantial psychological, social, and economic consequences, with a global prevalence estimated at approximately 1.4% and a markedly higher incidence among men (Tran et al., 2024). Although clinical research has traditionally focused almost exclusively on individuals diagnosed with GD, accumulating evidence over recent years consistently indicates that the negative consequences of gambling are not confined to the individual, but extend to family members and close social networks, exerting a significant and enduring impact (Li et al., 2017; Riley et al., 2021; Tulloch et al., 2022).

Within this context, gambling-related harm affects with particular intensity close family members and partners who play a central role in the patient’s emotional and social environment. Accordingly, different terms have been used in the literature to describe these individuals, depending on the theoretical framework and research setting. In particular, clinical and family-based models of addiction preferentially use the term Affected Others (AOs) to refer to partners, parents, or close relatives whose lives are directly impacted by the disorder and who are exposed to sustained stress, emotional strain, and relational disruption (Orford et al., 2017). This conceptualization is consistent with stress–strain–coping frameworks and has been widely adopted in clinical research examining the psychological burden and coping responses of family members affected by gambling-related problems (Dowling et al., 2025a; Tulloch et al., 2025).

In the present study, the term AOs is used to describe close family members or partners who accompanied patients with Gambling Disorder when accessing specialized treatment services, thereby representing a clinically relevant subgroup of individuals directly affected by gambling-related harm.

A growing body of research demonstrates that AOs experience substantial adverse outcomes. Psychological distress is common, including elevated rates of depression, anxiety, emotional dysregulation, substance use, and, in severe cases, suicidal ideation (Salonen et al., 2016; Castrén et al., 2021). Financial strain related to gambling losses often exacerbates stress, undermines family stability, and contributes to interpersonal conflict, social withdrawal, and relational breakdown (Langham et al., 2015; Downs and Woolrych, 2010). Physical health problems have also been reported among affected relatives, suggesting a cumulative burden associated with chronic exposure to gambling-related stress (Wenzel et al., 2008; Landon et al., 2018).

The degree and nature of harm experienced by affected others appears to vary according to relational proximity and role. Intimate partners, most frequently women, tend to report the highest levels of emotional burden, psychological distress, and relational harm, reflecting both greater exposure to gambling-related consequences and a higher likelihood of assuming caregiving or financial management roles (Tulloch et al., 2022; Goodwin et al., 2017; Tulloch et al., 2023).

From a theoretical perspective, the experience of affected others can be conceptualized within stress–strain–coping frameworks originally developed in the field of addiction research. These models propose that chronic exposure to a relative’s addictive behavior generates sustained stress, leading to psychological strain, the impact of which is moderated by available coping strategies and social support (Orford et al., 2017). Recent large-scale reviews have reinforced this perspective, showing that coping style plays a central role in shaping mental health outcomes among affected family members of individuals with gambling problems. In particular, maladaptive coping strategies, such as avoidance, self-blame, emotional suppression, and social withdrawal, have been consistently associated with greater psychological distress, whereas problem-solving, emotional expression, and support-seeking behaviors appear to confer a protective effect (Booth et al., 2021; Tulloch et al., 2025; Dowling et al., 2025a,b).

Research indicates that AOs employ various strategies to cope with the challenges of GD. Many use positive coping strategies, such as setting clear boundaries or issuing financial warnings to encourage accountability (Dowling et al., 2021) Other strategies, however, involve avoiding conflict by protecting the GD-affected individual’s reputation with friends and family, which can inadvertently enable the disorder (Ejova and Ohtsuka, 2020; Côté et al., 2022).

Although evidence suggests that involving family members in GD treatment improves adherence and outcomes for patients (Tremblay et al., 2018; Jiménez-Murcia et al., 2017), interventions continue to focus predominantly on the individual with GD, often overlooking the psychological needs and distress of affected AOs. Emerging interventions targeting AOs, including individual, couple-based, and online cognitive-behavioral approaches, emphasize the importance of addressing maladaptive coping patterns and strengthening adaptive strategies to reduce distress and improve overall family functioning (Magnusson et al., 2019; Edgren et al., 2022).

Lastly, to effectively support AOs, it is essential to provide them with information on GD, including its classification as a mental health disorder, as well as guidance on positive coping strategies (Salonen et al., 2014; Hing et al., 2022). Mental health professionals, particularly nurses, play a critical role in the detection, diagnosis, and intervention of both individuals with GD and their AOs. Effective involvement of AOs in the therapeutic process not only addresses their needs but may also improve treatment outcomes for those with GD (del Pino-Gutiérrez et al., 2017).

In light of these considerations, and given the scarcity of clinical studies focusing on coping strategies among affected family members who access treatment services together with individuals with GD, the present study aims to contribute empirical evidence from a specialized gambling treatment setting. Specifically, this study aims: (1) To describe the sociodemographic profiles of the sample of both patients diagnosed with GD and AOs. (2) To compare the sociodemographic and clinical profiles of AOs based on their kinship with patients and gender; (3) To analyze the association of coping strategies and AOs’ clinical profiles, in relation to kinship. (4) To explore the predictive model for the global psychological distress of AOs on their coping strategies and impulsivity (measured through the total score of the UPPS-P) and the patient’s character trait of self-direction.

## 2 Methodology

### 2.1 Participants

All patients were diagnosed with GD according to DSM-5 criteria (American Psychiatric Association, 2013) and were consecutively recruited from a specialized tertiary care unit. Each patient was asked to identify one close family member or partner who was most involved in their current situation to participate in the study as a AOs. The AOs group consisted of 184 individuals, corresponding to one AOs per patient. This procedure resulted in a clinically selected sample of affected family members actively involved in treatment, rather than a representative sample of all individuals affected by someone else’s gambling.

Exclusion criteria for patients included severe cognitive impairment, active psychosis, or acute suicidal ideation that could interfere with assessment reliability. AOs were excluded if they met diagnostic criteria for Gambling Disorder themselves. None of the AOs met exclusion criteria.

Participation was voluntary for both patients and AOs. Eligible dyads were invited consecutively during the recruitment period. No patient or AOs declined participation once invited, and all provided written informed consent.

### 2.2 Procedure

Participants were recruited from the Behavioral Addictions Unit at Bellvitge University Hospital (Barcelona, Spain), a tertiary referral center serving a population of over 2 million inhabitants.

Following the initial clinical intake interview, patients underwent a comprehensive diagnostic and clinical assessment conducted by licensed clinical psychologists with more than 20 years of experience in the field of gambling disorder. After diagnostic confirmation, patients completed an extensive battery of psychometric assessments and were invited to identify a close family member or partner to participate in the study as a AOs.

AOs received the study information, informed consent, and assessment materials through the patient and completed the questionnaires independently at home. Once returned, AOs questionnaires were reviewed by the clinical team to rule out the presence of GD in the relative. Subsequently, AOs received individualized feedback focused on their psychological profile, as part of the routine clinical assessment process.

AOs did not undergo a formal psychiatric diagnostic interview. Instead, psychological distress and symptom severity were assessed using the SCL-90-R, a widely used measure of general psychopathology. While AOs were screened to rule out Gambling Disorder, other psychiatric diagnoses were not systematically assessed, and therefore pre-existing psychiatric vulnerability cannot be ruled out.

Data collection and management were carried out by trained clinical psychologists and specialized mental health nurses.

### 2.3 Measures

Individuals diagnosed with GD, along with their respective AOs, underwent a comprehensive assessment battery that included the following psychometric instruments:

Completed by Patients and AOs:

Diagnostic Questionnaire for Gambling Disorder (according to DSM criteria) (Stinchfield, 2003). This 19-item self-report questionnaire is designed to assess the presence of GD by examining behavior over the past 12 months, following DSM criteria. It provides diagnostic information according to both DSM-IV-TR (2000) and DSM-5 (2013) standards. The Spanish psychometric adaptation of this tool has shown satisfactory reliability, with a Cronbach’s alpha of 0.81 (Jiménez-Murcia et al., 2009).

Symptom Checklist-Revised (SCL-90-R) (Derogatis, 1994). This self-report questionnaire is designed to evaluate psychological wellbeing through 90 items organized into nine primary (first order) dimensions: somatization, obsessive-compulsive behavior, interpersonal sensitivity, depression, anxiety, hostility, phobic anxiety, paranoid ideation, and psychoticism. Additionally, it includes three global indices: the global severity index (GSI), which combines the number and severity of symptoms as an indicator of psychological distress; the positive symptom total (PST), reflecting the count of endorsed items; and the positive symptom distress index (PSDI), which assesses the response style of patients who may exaggerate or minimize their psychological distress. The Spanish psychometric adaptation of this tool has shown satisfactory results, with a mean Cronbach’s alpha of *α* = 0.75 (González de Rivera et al., 1989).

Temperament and Character Inventory-Revised (TCI-R) (Cloninger, 1999). The TCI-R is a validated and reliable questionnaire consisting of 240 items designed to assess seven dimensions of personality. These include four temperament traits (novelty seeking, harm avoidance, reward dependence, and persistence) and three-character traits (self-directedness, cooperativeness, and self-transcendence). Responses are recorded on a 5-point Likert scale. The Spanish version demonstrated good internal consistency, with an average Cronbach’s alpha of 0.87 (Gutiérrez-Zotes et al., 2004).

Completes by patients:

Difficulties in Emotion Regulation Scale (DERS) (Gratz and Roemer, 2004). This self-report questionnaire comprises 36 items specifically designed to evaluate challenges in emotion regulation. It includes six primary subscales: (a) non-acceptance of emotional responses, (b) difficulties in maintaining goal-oriented actions, (c) issues with impulse control, (d) reduced emotional awareness, (e) limited access to effective emotion regulation strategies, and (f) low emotional clarity. An overall score is also provided, serving as a comprehensive index of emotional dysregulation. For this study, the Spanish adaptation was used, which has shown solid psychometric properties (Hervás and Jódar, 2008). The consistency of the questionnaire ranged from *α* = 0.750 to α = 0.933.

South Oaks Gambling Severity Screen (SOGS) (Lesieur and Blume, 1987). This diagnostic questionnaire consists of 20 items designed to evaluate the severity of gambling disorder. It effectively discriminates among probable pathological gamblers, problem gamblers, and individuals without gambling-related difficulties. The Spanish version of the questionnaire has demonstrated high reliability validity and internal consistency (Cronbach’s *α* = 0.94) (Echeburúa et al., 1994).

Impulsive Behavior Scale (UPPS-P) (Whiteside et al., 2005). The UPPS-P is a 59-item questionnaire designed to assess five distinct impulsivity traits: Lack of Perseverance, Lack of Premeditation, Sensation Seeking, Negative Urgency, and Positive Urgency. The Spanish version of this instrument has demonstrated strong reliability (Cronbach’s α ranging from 0.79 to 0.93) (Verdejo-García et al., 2010).

Completes by AOs.

Coping Strategies Inventory (CSI) (Tobin et al., 1989). Was used to assess coping styles. The original scale consisted of 72 self-administered items. In its Spanish adaptation, the scale was reduced to 40 items (Cano et al., 2007). The Spanish version demonstrates good psychometric properties, with Cronbach’s alpha values ranging from 0.75 to 0.89. The revised scale is organized into eight primary subscales: problem-solving, cognitive restructuring, social support, emotional expression, problem avoidance, wishful thinking, social withdrawal, and self-blame. These primary subscales are grouped into four secondary dimensions: problem-focused engagement, emotion-focused engagement, problem-focused disengagement, and emotion-focused disengagement, and two tertiary dimensions: engagement and disengagement.

For clarity, the three subscales most relevant to this study are defined as follows: *Problem-solving*: Active efforts to identify solutions and take steps to resolve the stressful situation. *Self-blame* (self-criticism): Negative self-evaluation and attributing responsibility for the problem to oneself. *Social withdrawal*: Avoidance of social interactions and isolation as a response to stress.

In addition, additional demographic, clinical, and social variables related to gambling were collected in the patient group through a semi-structured clinical interview. Regarding the AOs, sociodemographic information was obtained, including their relationship degree with the patient with gambling disorder, sex, age, education level, profession, current work activity, and marital status.

### 2.4 Statistical analysis

Data analysis was done with Stata18 for Windows. The comparison between the groups (defined for the relationships between AOs and the patients and the caregiver’s sex) was done with chi-square tests (X2) for categorical variables between the groups and with analysis of variance (ANOVA) for the quantitative features. These procedures included the calculation of the effect size through the Cramer’s-V for the contingency tables and Cohen’s-d for the mean differences. It was considered mild–moderate effect size for |*d|* > 0.50 or CV > 0.15, and high-large effect size for |*d*| > 0.80 or CV > 0.50 (Cohen, 1988; Kelley and Preacher, 2012). Additionally, control in the increase of Type-I error due to the use of multiple significance tests was done with Finner’s procedure (Finner and Roters, 2001).

Partial correlations adjusted by the covariates of AOs sex and age measured the association between the coping strategies and the clinical profile. Due to strong dependence between statistical significance for the R-coefficients with the sample size, effect size is interpreted as: low-poor for |*R*| > 0.10, moderate-medium for |*R*| > 0.24, and large-high for |*R*| > 0.37 (these thresholds correspond to Cohen’s-d of 0.20, 0.50 and 0.80 respectively) (Rosnow and Rosenthal, 1996).

Multiple linear regression was used to obtain an exploratory predictive model of the AOs global psychopathology distress (as measured with the SCL-90R GSI). The list of the potential predictors included the caregiver’s sociodemographic profile (sex, age, marital status and social position), the relationship between AOS and the patient (parents, couples, other), the caregiver’s capacity in coping strategies (CSI scales), the patients’ sociodemographic profile (sex, age, marital status and social position), the patients’ gambling measures (duration of the GD, DSM-5 total criteria, SOGS total, and gambling preference), and the patients’ clinical profile (SCL-90R GSI, UPPS-P total, DERS total, and TCI-R scales). The selection of the best predictors (variables that achieved significant contribution on the criterion) was done through stepwise procedure, consistent with the exploratory nature of the analysis.

### 2.5 Ethical approval

The study procedures adhered to the ethical principles outlined in the Declaration of Helsinki. Approval for the study was obtained from the University Hospital Clinical Research Ethics Committee (reference number PR203/19). All participants received detailed information about the study and provided written informed consent prior to their inclusion.

## 3 Results

### 3.1 Descriptive for the sample

Table 1 displays the descriptive for the sample of AOs. (First block) and the sample of patients (second block). Most AOs were women, married or living in a couple, with secondary education level, employed, and pertained to mean-low to low social position indexes. The distribution of the relationship between the AOs and the patients was: 40.8% parents, 38.6% spouses-couples, and 20.7% other. Most patients were men, single, with primary or secondary education levels, employed, and pertained to mean-low to low social position indexes. The distribution of the gambling preference of patients was: 40.8% non-strategic, 39.7% strategic and 19.6% mixed.

**Table 1:** Descriptive for the sample.

	AOs			Patients	
	*n*	*%*		*n*	*%*
Gender female	149	81.0%	Gender female	11	6.0%
Male	35	19.0%	Male	173	94.0%
Marital status single	40	21.7%	Marital status Single	93	50.5%
Married—couple	119	64.7%	Married—couple	71	38.6%
Divorced—separated	25	13.6%	Divorced—separated	20	10.9%
Education primary	52	28.3%	Education primary	85	46.2%
Secondary	91	49.5%	Secondary	79	42.9%
University	41	22.3%	University	20	10.9%
Employment unemployed	56	30.4%	Employment unemployed	66	35.9%
Employed	128	69.6%	Employed	118	64.1%
Social position mean-high to high	15	8.2%	Social position mean-high to high	25	13.6%
Mean	14	7.6%	Mean	22	12.0%
Mean-low	73	39.7%	Mean-low	52	28.3%
Low	82	44.6%	Low	85	46.2%
AOs parents	75	40.8%	Gambling non-strategic	75	40.8%
Couple	71	38.6%	Strategic	73	39.7%
Other	38	20.7%	Mixed	36	19.6%
	Mean	SD		Mean	SD
Age (years-old)	49.86	12.34	Age (years-old)	39.03	14.47

SD, standard deviation.

### 3.2 Comparison between groups

Table 2 includes the comparison of the AOs sociodemographic and clinical profiles based on their relationship with the patients. The group of AOs who were spouses/couples included the highest proportion of females and the highest mean score in the SCL-90R psychotic scale, while the group of AOs who were parents included the highest proportion of unemployed individuals, the highest proportion of subjects in the low social position index and the highest chronological mean age.

**Table 2:** Comparison of the AOs sociodemographic and clinical profiles based on their relationship with the patients.

	Parents (*n* = 75)		Couples (*n* = 71)		Others (*n* = 38)		Parents vs. couples		Parents vs. others		Couples vs. others	
	*n*	%	*n*	%	*n*	%	*p*	|CV|	*p*	|CV|	*p*	|CV|
Gender Female	54	72.0%	67	94.4%	28	73.7%	<0.001*	0.297†	0.850	0.018	0.002*	0.295†
Male	21	28.0%	4	5.6%	10	26.3%						
Marital status single	17	22.7%	9	12.7%	14	36.8%	<0.001*	0.338†	0.122	0.193†	0.003*	0.326†
Married – couple	40	53.3%	59	83.1%	20	52.6%						
Divorced – separated	18	24.0%	3	4.2%	4	10.5%						
Education primary	26	34.7%	16	22.5%	10	26.3%	0.270	0.134	0.515	0.108	0.700	0.081
Secondary	33	44.0%	37	52.1%	21	55.3%						
University	16	21.3%	18	25.4%	7	18.4%						
Employment unemployed	34	45.3%	15	21.1%	7	18.4%	0.002*	0.256†	0.005*	0.264†	0.737	0.032
Employed	41	54.7%	56	78.9%	31	81.6%						
Social mean-high to high	5	6.7%	6	8.5%	4	10.5%	0.025*	0.253†	0.222	0.197	0.771	0.102
Mean	2	2.7%	8	11.3%	4	10.5%						
Mean-low	26	34.7%	33	46.5%	14	36.8%						
Low	42	56.0%	24	33.8%	16	42.1%						

	Mean	SD	Mean	SD	Mean	SD	*p*	|*d*|	*p*	|*d*|	*p*	|*d*|
Age (years-old)	58.44	8.36	44.54	10.87	42.89	11.71	<0.001*	1.43†	<0.001*	1.53†	0.421	0.15
SCL-90R somatization	0.79	0.68	0.93	0.68	0.80	0.73	0.212	0.21	0.916	0.02	0.356	0.18
SCL-90R obsess.-comp.	0.96	0.55	1.16	0.74	1.01	0.74	0.067	0.31	0.683	0.08	0.267	0.20
SCL-90R Inter.sensitivity	0.72	0.60	0.90	0.60	0.67	0.62	0.068	0.31	0.675	0.08	0.056	0.38
SCL-90R depression	0.72	0.63	0.89	0.61	0.75	0.64	0.107	0.27	0.819	0.04	0.270	0.22
SCL-90R anxiety	0.65	0.57	0.80	0.63	0.66	0.54	0.125	0.25	0.924	0.02	0.241	0.24
SCL-90R hostility	0.56	0.59	0.66	0.60	0.64	0.61	0.313	0.17	0.500	0.13	0.870	0.03
SCL-90R phobic anxiety	0.49	0.54	0.68	0.63	0.54	0.64	0.066	0.31	0.705	0.08	0.253	0.22
SCL-90R paranoia	0.43	0.48	0.53	0.51	0.48	0.50	0.195	0.22	0.565	0.12	0.617	0.10
SCL-90R psychotic	0.52	0.44	0.71	0.63	0.59	0.62	0.042*	0.35	0.551	0.12	0.276	0.20
SCL-90R GSI	0.66	0.50	0.82	0.57	0.69	0.59	0.072	0.31	0.743	0.07	0.244	0.22
SCL-90R PST	33.21	17.90	38.85	18.27	35.11	22.73	0.077	0.31	0.620	0.09	0.332	0.18
SCL-90R PSDI	1.58	0.52	1.74	0.52	1.54	0.51	0.070	0.30	0.651	0.09	0.053	0.39

SD, standard deviation. *Bold: significant comparison (*p* < 0.05). †Bold: effect size in the ranges mild–moderate to high-large (|*d*| > 0.50 or CV>0.15).

Considering the AOs sex, differences were found in the relationship with the patients and the social position index (Table 3): most male AOs were parents (while most female AOs were spouses/couples) and pertained to higher social position index. Male AOs were also characterized, compared to women, for being older and reporting better psychopathology state.

**Table 3:** Comparison of the AOs sociodemographic and clinical profiles based on their gender.

	Women (*n* = 149)		Men (*n* = 35)			
	*n*	%	*n*	%	*p*	|CV|
AOs parents	54	36.2%	21	60.0%	0.001*	**0.271** ^**†**^
Couples	67	45.0%	4	11.4%		
Others	28	18.8%	10	28.6%		
Marital status single	33	22.1%	7	20.0%	0.785	0.051
Married – couple	97	65.1%	22	62.9%		
Divorced – separated	19	12.8%	6	17.1%		
Education primary	45	30.2%	7	20.0%	0.263	0.121
Secondary	74	49.7%	17	48.6%		
University	30	20.1%	11	31.4%		
Employment unemployed	46	30.9%	10	28.6%	0.790	0.020
Employed	103	69.1%	25	71.4%		
Socioeconómico mean-high to high	8	5.4%	7	20.0%	0.041*	**0.212** ^**†**^
Mean	12	8.1%	2	5.7%		
Mean-low	60	40.3%	13	37.1%		
Low	69	46.3%	13	37.1%		

	Mean	SD	Mean	SD	*p*	|*d*|
Age (years-old)	48.97	12.01	53.69	13.19	0.041*	0.37
SCL-90R somatization	0.91	0.69	0.59	0.63	0.012*	**0.51** ^**†**^
SCL-90R obsess.-comp.	1.10	0.67	0.82	0.65	0.029*	0.42
SCL-90R interpersonal sensitivity	0.85	0.61	0.49	0.52	0.002*	**0.63** ^**†**^
SCL-90R depression	0.86	0.63	0.53	0.51	0.006*	**0.56** ^**†**^
SCL-90R anxiety	0.76	0.60	0.49	0.48	0.015*	**0.50** ^**†**^
SCL-90R hostility	0.66	0.61	0.44	0.49	0.049*	0.39
SCL-90R phobic anxiety	0.63	0.61	0.36	0.53	0.016*	0.47
SCL-90R paranoid ideation	0.53	0.51	0.28	0.38	0.008*	**0.55** ^**†**^
SCL-90R psychotic	0.64	0.58	0.49	0.45	0.159	0.29
SCL-90R GSI	0.78	0.56	0.52	0.49	0.011*	**0.50** ^**†**^
SCL-90R PST	37.48	18.60	28.51	20.21	0.012*	0.46
SCL-90R PSDI	1.70	0.53	1.37	0.41	0.001*	**0.68** ^**†**^

SD, standard deviation. *Bold: significant comparison (0.05 level). ^†^Bold: effect size in the ranges mild–moderate to high-large (|*d*| > 0.50 or CV>0.15).

### 3.3 Correlation analysis

Table 4 contains the correlation matrix obtained in this work. Among the total sample, statistically significant associations were found between the CSI problem-solving, self-criticism, and social withdrawal scales and the psychopathology state (greater difficulties in coping strategies correlated with a worse psychological profile). This pattern was replicated depending on the relationship between the AOs and the patients (parents, spouses/couples, and others), with specific differences among each subsample. The highest number of statistically significant correlations (and the largest effect sizes for the coefficients) was reported for AOs, differing from parents and spouses/couples.

**Table 4:** Association between the AOs coping strategies and the clinical profile: partial correlations adjusted by relationship.

↓CSI SCL-90R → ®	SOM	OC	IS	DEP	ANX	HOS	PHO	PAR	PSY	GSI	PST	PSDI
Total sample (*n* = 184)												
Problems solving	**−0.308** ^**†**^	**−0.333** ^**†**^	**−0.330** ^**†**^	**−0.282** ^**†**^	**−0.310** ^**†**^	−0.192	**−0.285** ^**†**^	**−0.258** ^**†**^	**−0.267** ^**†**^	**−0.326** ^**†**^	**−0.373** ^**†**^	−0.210
Self-criticism	**0.383** ^**†**^	**0.305** ^**†**^	**0.295** ^**†**^	**0.338** ^**†**^	**0.404** ^**†**^	**0.377** ^**†**^	**0.326** ^**†**^	**0.299** ^**†**^	**0.427** ^**†**^	**0.383** ^**†**^	**0.374** ^**†**^	0.174
Emotion expression	0.007	0.055	−0.006	0.038	0.046	−0.045	0.097	−0.006	0.038	0.029	−0.079	0.057
Desiderata thinking	0.020	−0.031	−0.072	0.026	−0.009	0.057	−0.042	−0.037	0.023	−0.015	−0.078	0.082
Social support	−0.195	−0.186	−0.193	−0.168	−0.165	−0.120	−0.121	−0.142	−0.193	−0.192	−0.214	−0.152
Cognitive restructure	−0.061	−0.065	−0.053	−0.086	−0.022	−0.020	−0.074	−0.046	−0.015	−0.059	−0.056	−0.037
Problems avoidance	0.212	0.171	0.218	0.158	0.216	0.154	0.209	0.165	0.188	0.209	0.198	0.110
Social withdrawal	**0.424** ^**†**^	**0.401** ^**†**^	**0.386** ^**†**^	**0.376** ^**†**^	**0.401** ^**†**^	**0.309** ^**†**^	**0.349** ^**†**^	**0.385** ^**†**^	**0.375** ^**†**^	**0.423** ^**†**^	**0.393** ^**†**^	**0.360** ^**†**^
Parents (*n* = 75)												
Problems solving	−0.184	−0.153	**−0.248** ^**†**^	−0.114	−0.171	−0.093	−0.209	−0.101	−0.223	−0.191	**−0.244** ^**†**^	−0.110
Self-criticism	**0.263** ^**†**^	0.188	0.187	**0.264** ^**†**^	**0.293** ^**†**^	**0.256** ^**†**^	**0.259** ^**†**^	0.162	**0.353** ^**†**^	**0.282** ^**†**^	**0.327** ^**†**^	0.070
Emotion expression	−0.041	0.005	−0.008	0.020	0.049	−0.081	0.013	0.021	−0.057	−0.010	−0.105	0.063
Desiderata thinking	0.130	0.167	0.016	0.193	0.115	0.197	0.095	0.054	0.141	0.134	0.093	0.199
Social support	−0.228	−0.146	−0.234	−0.108	−0.127	−0.177	−0.140	−0.088	−0.214	−0.187	−0.202	−0.129
Cognitive restructure	0.070	0.138	0.051	0.071	0.196	0.075	0.067	0.026	0.093	0.105	0.087	0.089
Problems avoidance	0.140	0.119	0.167	0.111	0.185	0.069	0.130	0.138	0.122	0.158	0.165	0.060
Social withdrawal	**0.243** ^**†**^	0.182	**0.241** ^**†**^	0.226	**0.246** ^**†**^	0.180	**0.270** ^**†**^	**0.295** ^**†**^	0.220	**0.271** ^**†**^	**0.254** ^**†**^	0.201
Couples (*n* = 71)												
Problems solving	**−0.251** ^**†**^	**−0.315** ^**†**^	**−0.284** ^**†**^	**−0.247** ^**†**^	**−0.257** ^**†**^	−0.027	−0.171	−0.134	−0.096	**−0.244** ^**†**^	**−0.288** ^**†**^	−0.148
Self-criticism	**0.443** ^**†**^	**0.366** ^**†**^	**0.391** ^**†**^	**0.372** ^**†**^	**0.499** ^**†**^	**0.488** ^**†**^	**0.302** ^**†**^	**0.317** ^**†**^	**0.462** ^**†**^	**0.434** ^**†**^	**0.393** ^**†**^	**0.301** ^**†**^
Emotion expression	0.217	**0.330** ^**†**^	0.137	0.212	0.203	0.172	**0.313** ^**†**^	0.156	**0.281** ^**†**^	**0.250** ^**†**^	0.147	**0.256** ^**†**^
Desiderata thinking	0.026	0.026	−0.002	−0.008	0.036	0.116	0.032	0.058	0.038	0.022	−0.068	0.152
Social support	−0.131	−0.115	−0.098	−0.157	−0.085	0.013	−0.028	−0.067	−0.095	−0.110	−0.173	−0.010
Cognitive restructure	−0.193	−0.227	−0.186	**−0.293** ^**†**^	−0.210	−0.069	−0.190	−0.091	−0.071	−0.209	−0.230	−0.159
Problems avoidance	0.156	0.062	0.119	0.100	0.115	0.056	0.110	0.028	0.087	0.100	0.051	0.049
Social withdrawal	**0.374** ^**†**^	**0.360** ^**†**^	**0.341** ^**†**^	**0.337** ^**†**^	**0.370** ^**†**^	0.202	0.197	0.237	0.232	**0.327** ^**†**^	**0.273** ^**†**^	**0.326** ^**†**^
Others (*n* = 38)												
Problems solving	**−0.590** ^**†**^	**−0.612** ^**†**^	**−0.541** ^**†**^	**−0.604** ^**†**^	**−0.662** ^**†**^	**−0.573** ^**†**^	**−0.536** ^**†**^	**−0.726** ^**†**^	**−0.631** ^**†**^	**−0.653** ^**†**^	**−0.645** ^**†**^	**−0.525** ^**†**^
Self-criticism	**0.371** ^**†**^	**0.279** ^**†**^	0.177	**0.314** ^**†**^	**0.331** ^**†**^	**0.298** ^**†**^	**0.396**	**0.444** ^**†**^	**0.472** ^**†**^	**0.362** ^**†**^	**0.331** ^**†**^	0.076
Emotion expression	**−0.247** ^**†**^	**−0.364** ^**†**^	−0.238	−0.197	**−0.288** ^**†**^	**−0.359** ^**†**^	−0.171	**−0.269** ^**†**^	−0.226	**−0.271** ^**†**^	**−0.368** ^**†**^	**−0.303** ^**†**^
Desiderata thinking	−0.197	**−0.352** ^**†**^	**−0.360** ^**†**^	−0.172	**−0.294** ^**†**^	**−0.255** ^**†**^	**−0.284** ^**†**^	**−0.348** ^**†**^	−0.170	**−0.289** ^**†**^	**−0.309** ^**†**^	**−0.242** ^**†**^
Social support	**−0.308** ^**†**^	**−0.366** ^**†**^	**−0.293** ^**†**^	**−0.275** ^**†**^	**−0.444** ^**†**^	**−0.373** ^**†**^	**−0.307** ^**†**^	**−0.299** ^**†**^	**−0.280** ^**†**^	**−0.347** ^**†**^	**−0.338** ^**†**^	**−0.446** ^**†**^
Cognitive restructure	−0.160	−0.132	−0.082	−0.080	−0.167	−0.163	−0.133	−0.114	−0.088	−0.130	−0.067	−0.151
Problems avoidance	**0.414** ^**†**^	**0.426** ^**†**^	**0.488** ^**†**^	**0.346** ^**†**^	**0.474** ^**†**^	**0.442** ^**†**^	**0.525** ^**†**^	**0.472** ^**†**^	**0.511** ^**†**^	**0.481** ^**†**^	**0.458** ^**†**^	**0.305** ^**†**^
Social withdrawal	**0.722** ^**†**^	**0.670** ^**†**^	**0.616** ^**†**^	**0.606** ^**†**^	**0.681** ^**†**^	**0.691** ^**†**^	**0.729** ^**†**^	**0.706** ^**†**^	**0.756** ^**†**^	**0.733** ^**†**^	**0.705** ^**†**^	**0.604** ^**†**^

SCL-90R scales: SO, Somatization; OC, Obsessive-compulsive; IS, Interpersonal sensitivity; DEP, Depression; ANX, Anxiety; HOS, Hostility; PHO, Phobic anxiety; PAR, Paranoid ideation; PSY, Psychotic; GSI, PST, PSDI, global indexes. ^†^Bold: effect size in the range mild–moderate to high-large (|*R*| > 0.24).

### 3.4 Predictive model

The predictive model for the AOs global psychology distress (SCL-90R GSI) is displayed in Table 5. The worse psychopathology state was associated to AOs with more difficulties in the coping strategies (concretely in problem solving, self-criticism, emotion expression, social support and social withdrawal), who take care of patients with lower impulsivity levels and lower TCI-R self-directedness scores. The global predictive capacity of this model, as measured with the adjusted R-squared coefficient, was around 30%.

**Table 5:** Predictive model of the AOs global psychology distress (SCL-90R GSI).

CSI problems solving	*B*	*SE*	Beta	*p*	95% CI B	
	−0.029	0.011	−0.182	0.009	−0.051	−0.008
CSI self-criticism	0.028	0.008	0.250	<0.001	0.013	0.044
CSI emotion expression	0.019	0.008	0.155	0.026	0.002	0.035
CSI social support	−0.018	0.007	−0.176	0.011	−0.032	−0.004
CSI social withdrawal	0.033	0.009	0.250	<0.001	0.015	0.051
Patients’ impulsivity (UPPS total)	−0.004	0.002	−0.171	0.035	−0.008	0.000
Patients’ self-directedness	−0.005	0.002	−0.181	0.027	−0.009	−0.001
Adjusted R-squared coefficient: 0.301						

SE, standard error. 95%CI: 95% confidence interval.

## 4 Discussion

The results obtained in this study allowed us to describe the sociodemographic profiles of the sample, showing that most AOs were women, married or partnered, with secondary education, employed, or belonging to lower-middle or low socioeconomic strata. These findings are consistent with previous research indicating that GD primarily affects men, while women tend to assume the role of AOs (Hing et al., 2022; Lind et al., 2022; Jiménez-Murcia et al., 2020; Morelli et al., 2019).

Secondly, the comparison of sociodemographic and clinical profiles of AOs according to their relationship with patients and gender revealed that most male AOs were fathers, whereas most female caregivers were predominantly wives or partners. Furthermore, women exhibited worse psychopathological states than male AOs. These findings align with previous studies reporting that female AOs experience a greater emotional and psychological burden (Salonen et al., 2014). Additionally, the fathers’ group showed the highest proportion of unemployed individuals, the lowest social index position, and the highest mean age. Retirement among this subgroup may partially explain this pattern.

Regarding the relationship between AOs and patients with GD, parents were the predominant group, followed by partners. In our sample, nearly half of the patients were single, making parents the primary source of support during recovery. The absence of ex-partners in our clinical sample probably reflects the treatment-focused design, as ex-partners are less likely to assume caregiving roles or facilitate attendance at clinical services. In contrast, community-based studies consistently identify ex-partners as a commonly affected group (Tulloch et al., 2023; Hing et al., 2022), highlighting the importance of study context when comparing AOs profiles. The increase in parents as AOs may also be related to an increasingly younger age of gambling onset (Çıtak, 2023), a phenomenon facilitated by easy access to online gambling platforms, available anytime and anywhere through digital devices (Du et al., 2024). Another plausible explanation involves cross-cultural and economic factors; for instance, in Southern European countries, young adults tend to achieve independence later in life due to specific family dynamics and economic challenges, including difficulties accessing the labor market (Eurostat, 2026).

Moreover, this study indicates that coping strategies are strongly associated with psychological distress among AOs of individuals with GD. Specifically, higher levels of self-criticism and social withdrawal were linked to greater psychopathology, whereas problem-solving was associated with more favorable psychological profiles. These observations are consistent with previous evidence. First, Estevez et al. (2020) highlight that difficulties in emotion regulation and coping are common among AOs, which aligns with our finding that self-criticism and withdrawal correlate with worse psychological outcomes. Complementarily, Orford et al. (2017) emphasize the impact of stress and emotional strain, underscoring the role of social support as a buffer against distress; our results confirm that the absence of adaptive strategies, such as seeking support and problem-solving, is associated with poorer clinical indicators. Similarly, Chan et al. (2016) demonstrate that AOs coping strategies directly influence psychological adjustment and relational dynamics, which is reflected in our data: AOs using adaptive coping strategies exhibit better psychological states, whereas those relying on self-criticism and isolation show greater deterioration. Finally, Tulloch et al. (2025) stress the role of coping style and social support in harm experiences, which aligns with our findings that the lack of adaptive coping and support networks increases psychological distress. Collectively, these findings suggest that interventions should focus on reducing self-criticism and social withdrawal while strengthening problem-solving, emotional expression, and support-seeking—components essential for improving AOs wellbeing and indirectly facilitating patient recovery.

The comparative analysis of psychopathological states across AOs subgroups revealed that the group classified as “others” presented the worst clinical prognosis. This subgroup included individuals with greater difficulties in coping strategies (specifically problem-solving, self-criticism, emotional expression, social support, and social withdrawal), who were identified as AOs of patients with lower levels of impulsivity (UPPS-P) and self-directedness (TCI-R). Previous research suggests that AOs who are parents or partners typically experience greater direct responsibility and emotional involvement, implementing coping strategies more effectively. These characteristics favor treatment-seeking in both patients diagnosed with GD and AOs themselves, enabling better management of conflict situations (Chan et al., 2016; Khan and Kamal, 2019; Marzban et al., 2024).

Finally, it is worth noting that the global predictive capacity of our model was 30%. These results indicate that coping strategies and sociodemographic characteristics explain a significant portion of AOs’ psychopathological state. However, other factors not examined in this study—such as social support, financial difficulties, or physical health—may also influence mental health (Mikulić et al., 2023). Future research should explore these additional variables to refine predictive models. Moreover, future studies should assess whether enhancing adaptive coping—particularly problem-solving, cognitive restructuring, emotional expression, and social support—reduces psychological distress among AOs and improves treatment outcomes, ideally through controlled trials and community-based sampling that includes ex-partners and underrepresented groups.

### 4.1 Limitations

The study presents several limitations that must be considered on interpreting the findings. First of all, the sample was recruited from a center specialized in the treatment of gambling disorder, so the results should not be generalized in the general population. The selection of the AOs was based on the choice of the patients themselves, which could have introduced a bias on not including other members of the family or from the social environment, which could also be affected. Moreover, the use of self-administered questionnaires by the AOs could have generated certain errors associated with the interpretation of the items. AOsAos Finally, the predictive model of AOs’ psychological distress was developed using a stepwise regression approach and should be considered exploratory. Although the model accounted for approximately 30% of the variance, other relevant variables, such as perceived social support, financial strain, family conflict, or prior life events, were not included and may also contribute to AOs’ psychological wellbeing. Future research using theory-driven and longitudinal analytic approaches is warranted to refine predictive models.

## 5 Conclusion

The results of this study strengthen the consideration of GD as a public health problem that spreads to the patient and affects the family, working and social environment in a significant way. The socio-demographic characteristics of the AOs in this study has allowed us to identify relevant variables, such as the greater vulnerability of the women in the roles of partner or mother, which suggests the need to explore the gender differences even further in future research. In addition, the analysis of the coping strategies brings to light its fundamental role in the psychological wellbeing of the AOs. Strategies such as self-criticism, and social withdrawal have been associated with a greater psychopathological discomfort, whereas problem solving, the search for social support and emotional expression have been related to better results in the mental health of the AOs. These findings underline the importance of continuing to explore other contextual and family factors that can influence in the wellbeing of the AOs, like the analysis of life events. Besides, the predictive model developed has allowed us to identify variables in the appearance of psychological discomfort, highlighting the impact factors such as the coping strategy, social support, and specific psychological characteristics such as impulsivity and self-direction. In this way, the results obtained offer relevant clinical implications for the design of interventions directed toward the AOs, orientated to reduce their psychological discomfort and strengthen their coping skills, thus favoring a comprehensive approach in the treatment of GD (Edgren et al., 2022).

## Data Availability

In accordance with current regulations on data protection and research ethics, database queries must be conducted exclusively within the hospital service responsible for data custody and management. Requests to access the datasets should be directed to Susana Jiménez-Murcia: sjimenez@bellvitgehospital.ca.
